# Taxonomy, diversity, temporal and geographical distribution of Cutaneous Leishmaniasis in Colombia: A retrospective study

**DOI:** 10.1038/srep28266

**Published:** 2016-06-22

**Authors:** Juan David Ramírez, Carolina Hernández, Cielo M. León, Martha S. Ayala, Carolina Flórez, Camila González

**Affiliations:** 1 Grupo de Investigaciones Microbiológicas-UR (GIMUR), Programa de Biología, Facultad de Ciencias Naturales y Matemáticas, Universidad del Rosario, Bogotá-Colombia; 2 Grupo de Parasitología, Instituto Nacional de Salud, Bogotá-Colombia; 3 Centro de Investigaciones en Microbiología y Parasitología Tropica (CIMPAT), Universidad de Los Andes, Bogotá-Colombia

## Abstract

Leishmaniases are tropical zoonotic diseases, caused by kinetoplastid parasites from the genus *Leishmania*. New World (NW) species are related to sylvatic cycles although urbanization processes have been reported in some South American Countries such as Colombia. Currently, few studies show the relative distribution of *Leishmania* species related to cutaneous Leishmaniasis (CL) in South America due to the lack of accurate surveillance and public health systems. Herein, we conducted a systematic estimation of the *Leishmania* species causing CL in Colombia from 1980 to 2001 via molecular typing and isoenzymes. A total of 327 *Leishmania* isolates from humans, sandflies and reservoirs were typed as *L. panamensis* 61.3% (201), *L. braziliensis* 27.1% (88), *L. lainsoni* 0.6% (2), *L. guyanensis* 0.9% (3), *L. infantum chagasi* 4% (12), *L. equatoriensis* 0.6% (2), *L. mexicana* 2.1% (8), *L. amazonensis* 2.8% (9) and *L. colombiensis* 0.6% (2). This is the first report of two new *Leishmania* species circulating in Colombia and suggests the need to convince the Colombian government about the need to deploy and standardize tools for the species identification to provide adequate management to individuals suffering this pathology.

Leishmaniases include a spectrum of diseases caused by the flagellate protozoan *Leishmania*, an obligate intracellular protozoan parasite that infects humans and other mammals^1^. The clinical manifestations of the disease include cutaneous leishmaniasis (skin ulcers), mucocutaneous leishmaniasis, and visceral leishmaniasis (lethal spleen/liver inflammation); the parasite is transmitted to humans by the bite of infected sandflies (Psychodidae family)^2^. Leishmaniases are prevalent in 98 countries with an incidence of 1.3 million of new cases each year, although only half is reported. The visceral form causes 300,000 cases (90% in Bangladesh, Brazil, Ethiopia, India, Nepal, South Sudan and Sudan) and one million belong to the cutaneous (mostly in Afghanistan, Algeria, Brazil, Colombia, Iran, Pakistan, Peru, Saudi Arabia, Syria and Tunisia) or mucocutaneous forms (especially in Brazil, Peru and Bolivia)^1^. The genus *Leishmania* includes more than 20 species and is divided into 3 subgenera, according to the development site of the parasite in the sandfly: *Leishmania* (*Leishmania*), *Leishmania* (*Viannia*) and *Leishmania* (*Sauroleishmania*). Species and subspecies grouped in complexes are under constant review and debate with recent descriptions of new cryptic species^3^,^4^. The difficult classification of multiple species and subspecies of *Leishmania* depends on several aspects and species identification is needed to provide adequate treatment for infected patients: i) Biological: development in the sandfly, growth in culture media and development in vertebrate hosts^5^ ii) Biochemical: isoenzyme patterns, sequencing of multiple loci (multilocus enzyme typing)-current “gold standard”^5^,^6^ iii) Immunological: parasitic analysis with monoclonal antibodies^7^; iv) Phylogenetical: multilocus sequence typing and DNA sequencing of single/multiple loci^8^.

Cutaneous leishmaniasis (CL) is the most common form of leishmaniasis and causes ulcers on the exposed parts of the body, leaving scars for life. About 95% of CL cases occur in the Americas, the Mediterranean, the Middle East and Central Asia. More than two thirds of new cases of CL occur in six countries: Afghanistan, Algeria, Brazil, Colombia, Iran and Syria. An estimated of 0.7 million to 1.3 million new cases occur worldwide annually^1^,^2^,^9^. Although leishmaniasis is estimated to cause the greatest burden of disease among single infectious disease, it is ignored in discussions on priorities for tropical disease control^10^. This is due to its complex epidemiology and ecology, lack of simple and easy tools to apply for case management, and limited current incidence data^11^,^12^. Also, the variations of etiologic agents causing a number of different pathologies complicate the treatment, management and control of this tropical disease.

Colombia is one of the three countries with the highest number (seven species) of *Leishmania* parasites affecting humans^13^,^14^. About 99.3% of all cases are CL, 0.4% mucocutaneous leishmaniasis (MCL) and 0.3% visceral leishmaniasis (VL)^15^. The disease prevails in much of the country, moving from sylvatic to domestic cycles^16^. Cutaneous leishmaniasis outbreaks caused by *L. braziliensis*, *L. panamensis* and *L. guyanensis* are associated with intra and peridomiciliary transmission, which have been reported since 1984^17^. However, most studies in the country do not report the species involved and likewise have never assessed the genetic variability of the species circulating in the country.

From 1990 to 1999, 58897 cases were recorded in Colombia, 96.35% of CL, 2.65% of MCL and 1% of VL. Epidemiological data does not include species identification, and to date, data on parasite species distribution on a country scale is scarce. The aim of this study was to assess *Leishmania* distribution in Colombia based on parasite isolates from patients with CL, mammals, and sandfly vectors collected in different geographical regions of Colombia, and recruited in the National Leishmaniasis surveillance program from 1980 to 2001, using techniques unavailable at the time data were collected. The cytochrome b gene was sequenced in order to establish the circulating species, determine the genetic diversity and assessing its potential use in relation to the gold standard markers as MLEE.

## Materials and Methods

Intensive sampling was conducted during 21 years as part of the epidemiological surveillance of CL conducted by the National Health Institute in Colombia. Isolates from humans, sandflies and mammals from 22 departments in Colombia (Antioquia, Bolivar, Boyaca, Caldas, Caqueta, Casanare, Choco, Cordoba, Cundinamarca, Guainia, Guajira, Guaviare, Huila, Magdalena, Meta, Norte de Santander, Putumayo, Risaralda, Santander, Sucre, Tolima and Vichada) with high, medium and low endemicity were obtained. Sandflies (*Lutzomyia longipalpis, Pintomyia spinicrassa* and *Psathyromyia shannoni*) and mammals (*Canis lupus familiaris*, *Didelphis marsupialis* and *Akodon* sp.) were captured in domestic (within dwellings), peridomestic (near dwellings) and sylvatic (more than 250 meters from dwellings) localities. To perform mammal sampling, technicians previously trained by veterinarians took the punch biopsy; sampled species were not endangered or protected. In the case of domestic animals, the owners provided oral informed consent to allow the sampling. Animals were anesthetized and a punch biopsy was collected. In all cases, animals were released and manipulated following the international guiding principles for biomedical research involving animals, as issued by the Council for International Organizations of Medical Sciences. The methods were carried out in accordance with the above mentioned approved guidelines. All experimental protocols were approved by the ethics and technical scientific committee of National Institute of Health in Bogotá, Colombia.

*Leishmania* parasites were isolated from human patients performing methods that were carried out in accordance with the approved guidelines by the ethics and technical scientific committee of National Institute of Health in Bogotá, Colombia (All experimental protocols were approved by this institute´s committee). Written informed consent was obtained from all subjects.

### Leishmania Cytb barcoding and comparison with MLEE

We obtained 327 isolates (311 from humans, 6 from mammalian reservoirs and 10 from sandflies). Punch biopsies were triturated in sterile Ten Broeck homogenizers containing phosphate buffered saline (PBS), gentamicin (40 ug/ml), and 5-fluorocytosine (500 μg/mL). The resultant tissue suspension was inoculated directly into 2 tubes of NNN medium. The methods used for processing sand flies for parasite isolation were as reported elsewhere^18^. DNA was extracted from 200–μL aliquots of the exponential phase cultures using a QIAamp DNA Isolation Kit. The DNA quality and concentration were measured at 260 nm and stored at −20 °C.

The isolates included in this study were previously typed by Multilocus Enzyme Electrophoresis using distinct enzymes such as Lactate dehydrogenase (1.11.27), Malate dehydrogenase (1.1.1.37), Malic enzyme (1.1.1.40), Isocitrate dehydrogenase (1.1.1.42), Phosphogluconate dehydrogenase (1.1.1.44), Glucose-6-phosphate dehydrogenase (1.2.1.49), Glutathion reductase (e1.6.4.2), Glutamate-oxaloacetransaminase (2.6.1.1), Glutamate-pyruvate transaminase (2.6.1.2), Hexokinase (2.7.1.1) 6-Phosphofructokinase (2.7.1.11), Phosphoglucomutase (7.2.5.1), Acid phosphatase (3.1.3.2), Peptidase D (3.4.13.9), Fumarate hydratase (4.2.1.2), Mannose phosphate isomerase (5.3.1.8) and Glucose phosphate isomerase (5.3.1.9) following the specifications and reference strains reported elsewhere^19^. Additionally, the partial region of the cytochrome b gene (marker encoded on maxicircles) was amplified by PCR in a total volume of 50 uL containing 1X of Buffer, 100 mM of dNTPs, 50 pM of each primer, 5U of Taq Polymerase (Invitrogen) and 10 ng of DNA. The PCR products were digested with EXOSAP (Affymetrix, USA) and sequenced by the dideoxy-terminal method in an automated capillary sequencer (AB3730, Applied Biosystems) by both strands in Macrogen (Korea). The sequences were submitted to BLASTn for similarity search with *Leishmania* sequences deposited on the databases. Since Cytb marker is not able to discriminate *L. braziliensis* from *L. peruviana* species, those isolates typed as *L. braziliensis* were subsequently typed by direct sequencing of HSP70 as recommended elsewhere^20^,^21^.

### Phylogenetic reconstruction and diversity analyses

The resulting sequences were edited in MEGA 5.0 and aligned using ClustalW 1.8 with reference sequences from *L. donovani donovani* (AB095957), *L. donovani infantum* (AB095958), *L. donovani chagasi* (AB095959)*, L. tropica* (AB095960), *L. major* (AB095961), *L. aethiopica* (AB095962), *L. mexicana mexicana* (AB095963), *L. amazonensis* (AB095964), *L. garnhami* (AB095965), *L. braziliensis* (AB095966), *L. panamensis* (AB095968), *L. guyanensis* (AB095969), *L. equatoriensis* (AB434687), *L. pifanoi* (EF579907), *L. lainsoni* (AB433280), *L. colombiensis* (KF302738) and *L. peruviana* (AB433282) retrieved from GeneBank as suggested by different studies^22^,^23^. A maximum composite likelihood (MCL) analysis using a Tamura-3 parameter was run in RaxML Phylogeny.fr platform. To evaluate the robustness of the nodes in the resulting phylogenetic tree, 1000 bootstrap replicates were performed.

In addition to ML analyses, a Nexus matrix was constructed for haplotype network analysis in Network 2.0 using a median-joining model based on 1000 iterations with default parameters. The purpose of this analysis was to determine the diverse number of mitochondrial alleles across the population and determine the biological and geographical distribution of the alleles depicted for four species (*L. panamensis, L. braziliensis, L. amazonensis* and *L. infantum chagasi*). Lastly, sequence genetic diversity was estimated for Cytb gene fragment by species set. π and θ nucleotide diversity indexes and haplotype diversity were calculated in DNAsp v.5.0.

### Spatial, temporal and ecological distribution patterns

To address the spatial distribution of *Leishmania* parasites isolated, a georeferenced database was constructed. Data on human isolates belong to the health center where patients were diagnosed and location of insects and mammals belong to the capture locality. To assess temporal variation, the database was divided in four ranks and distribution maps for each time-set were built. Although this temporal variation does not reflect parasite dispersal, it does reunite all epidemiological information available in the country and allow us to describe potential distribution during the time of the study. For those parasite species with seven or more collection data points, ecological analyses were performed. Using ArcGIS10.3 we extracted values of Ecoregions^24^ and Colombian Ecosystems^25^ in order to describe parasite distribution by Ecosystems and land´s use coverage. Additionally, using vector species potential distribution maps, published by Ferro and collaborators in 2015, we described phlebotominae of medical importance in Colombia spatially associated to parasite occurrences.

## Results

### *Leishmania* Cytb barcoding and comparison with MLEE

A total of 327 *Leishmania* isolates were typed by means of Cytb barcoding and MLEE profiles. Edited sequences were submitted to Blastn search for sequence and hit similarity to conduct the Cytb barcoding, in general the sequences showed an average identity of 98% with the reported Genbank sequences. Overall, the concordance between MLEE and Cytb was high (Kappa Cohen Index 0.833 p < 0.05), a total of 19 (5.8%) isolates showed incongruences as follows: MLEE did not identify four isolates (Identity uncertain) that Cytb barcoding reported as *L. colombiensis* (2 isolates) and *L. equatoriensis* (2 isolates); two isolates were typed as *L. guyanensis* by MLEE and typed as *L. lainsoni* by Cytb. MLEE classified 2 isolates as *L. mexicana* that Cytb typed as *L. amazonensis*. Lastly, there were 11 strains that showed a hybrid profile *L. panamensis/L. braziliensis* by MLEE and typed as *L. panamensis* by Cytb.

**Figure 1:**
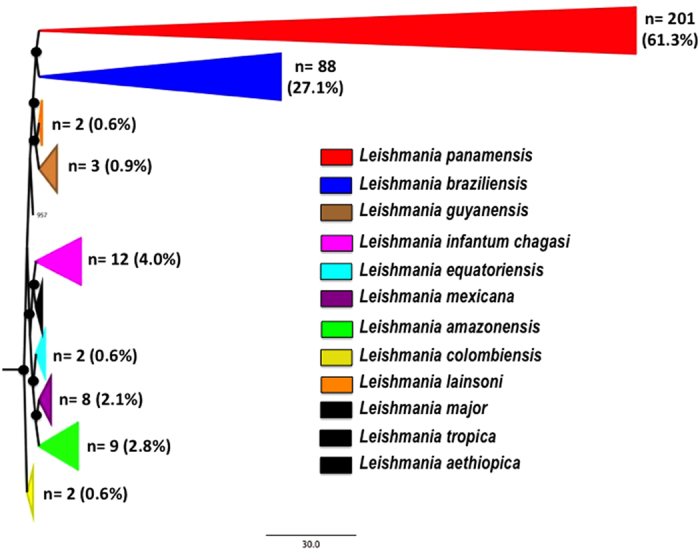
Maximum Composite Phylogenetic reconstruction of Cytb sequences. Phylogenetic reconstruction of 339 Cytb gene sequences obtained from the *Leishmania* isolates included in the study and the reference strains retrieved from GenBank. Black dots indicated bootstrap values equal or over 85%.

These incongruences were further confirmed by sequencing of HSP70 gene fragment where the Blastn hits showed concordance with the Cytb barcoding findings. Based on the final consensus of Cytb and HSP70 typing (Incongruent isolates and further typing of *L. braziliensis* isolates), the frequency of *Leishmania* species detected were as (Table S1): *L. panamensis* 61.3% (201), *L. braziliensis* 27.1% (88), *L. lainsoni* 0.6% (2), *L. guyanensis* 0.9% (3), *L. infantum chagasi* 4% (12), *L. equatoriensis* 0.6% (2), *L. mexicana* 2.1% (8), *L. amazonensis* 2.8% (9) and *L. colombiensis* 0.6% (2). The sequences were aligned and robust phylogenetic reconstruction was conducted observing concordance with the genetic clustering reported by other groups using Cytb marker and the independent barcoding of the samples. The ML unrooted tree showed high support bootstrap for the species identified across our dataset (Fig. 1).

### Phylogenetic reconstruction and diversity analyses

In total, the Cytb gene showed 82 polymorphic sites for all the species studied with a total of 87 mutations. The genetic diversity by species was measured showing that *L. panamensis* presented the highest genetic diversity followed by *L. infantum chagasi* and *L. braziliensis* based on the haplotype diversity index. *Leishmania guyanensis, L. amazonensis* and *L. colombiensis* showed moderate levels of haplotype diversity index. Lastly, *L. lainsoni* showed a tailored degree of haplotype diversity (Hd = 1) and *L. mexicana* and *L. equatoriensis* showed absence of haplotypic diversity (Hd = 0) (Table 1). When π and θ were observed, the pattern maintained as the observed with the haplotypic diversity index. In particular, *L. lainsoni* showed high values along these genetic diversity indexes.

**Figure 2:**
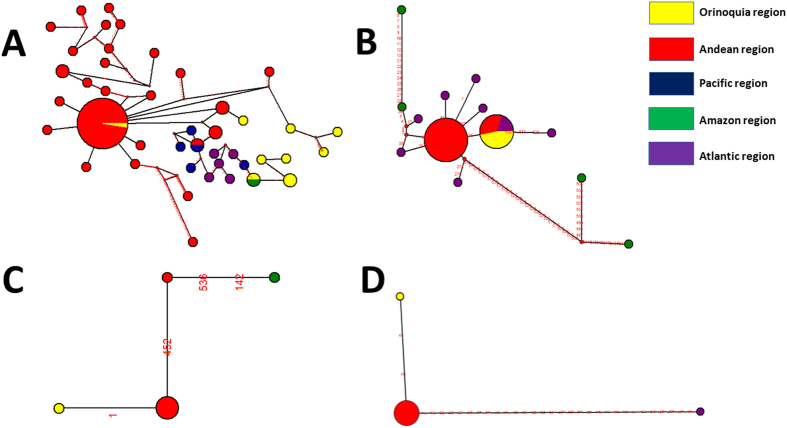
Network analysis of geographical distribution of Leishmania species. Alleles of the Cytb gene were retrieved to construct the networks shown for each species as follows, the numbers on the lines specify the positions across the alignment where a nucleotide change occurred (**A**) *L. panamensis*; (**B**) *L. braziliensis*; (**C**) *L. amazonensis*; (**D**) *L. infantum chagasi.*

Haplotype networks were constructed to determine the mitochondrial alleles distributed according to geography and host (human, sandfly, mammalian reservoir). These analyses were only conducted in *L. panamensis, L. braziliensis, L. infantum chagasi* and *L. amazonensis* isolates because were the most prevalent species across our study. We divided the department based on eco-geographical regions and observed that all four species alleles are circulating in the Andean region. *L. panamensis* was the most diverse species with one dense haplotype harboring alleles from Andean and Orinoquia region, a second haplotype harboring alleles from Andean and Pacific regions and a third haplotype harboring alleles from Orinoquia and Amazon region. The rest of the haplotypes were considered independent based on the geographical subdivisions. The genetic connectivity was illustrated and each geographical region is clustered with the exception of the Orinoquia region haplotypes that showed two genetic backgrounds (Andean region and Amazon region) (Fig. 2A). For *L. braziliensis*, there was one unique haplotype harboring different geographical regions alleles (Atlantic, Orinoquia and Andean regions); the rest of haplotypes were unique based on the geographical region. Curiously, Amazon region alleles are shared with the Andean and Atlantic regions (Fig. 2B). *L. infantum chagasi* showed three haplotypes with independent alleles from the Andean and Amazon regions and in the case of *L. amazonensis* three haplotypes with alleles from the Orinoquia, Andean and Atlantic regions (Fig. 2C,D). Network analyses were also conducted and colored based on the hosts; this analysis is limited due to the high number of isolates obtained from humans and the low number from sandflies and mammalian reservoirs. However, in all the cases (*L. panamensis, L. braziliensis, L. infantum chagasi* and *L. amazonensis*), it was possible to determine that alleles from the three hosts are shared confirming the maintenance of the transmission in the areas (Fig. 2).

### Spatial, temporal and ecological distribution patterns

**Figure 3:**
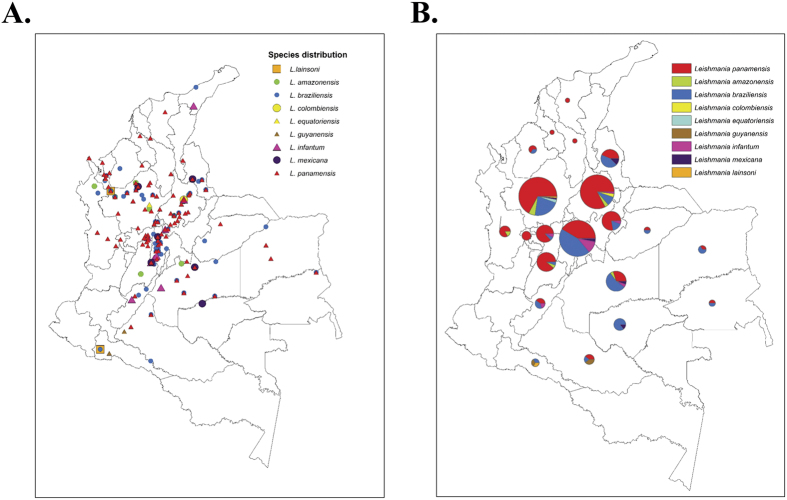
Geographical distribution of 327 *Leishmania* isolates associated to CL in Colombia. GPS coordinates were used to build georeferenced maps of isolates location. The maps were built on ArcGIS10.3 (http://www.esri.com/ArcGIS10.3) (**A**) Georeferenced isolates discriminated by species in the country. (**B)** Relative abundance of *Leishmania* species in each Colombian department.

The most widespread species in Colombia was *L. panamensis*, 201 isolates in 109 localities distributed across 19 departments (Fig. 3); its distribution ranges from lowlands to highlands in the Andean region. Similar patterns of geographical distribution were found for *Leishmania braziliensis*, isolated in 63 localities from 17 departments while *L. amazonensis* was isolated in nine localities widely distributed in five departments, and *L. infantum chagasi* in nine localities in six departments. Three departments showed the highest abundance of the isolates (Antioquia, Cundinamarca and Santander). Interestingly, Antioquia contributed the highest number of CL and ML cases, and only five departments contributed 52% of CL cases from 1990 to 1999. Poorly known species with few records were detected during the sampling: *L. lainsoni* (2 isolates), obtained from humans in Antioquia and Putumayo, and *L. equatoriensis* (2 isolates) from humans in Antioquia.

**Figure 4:**
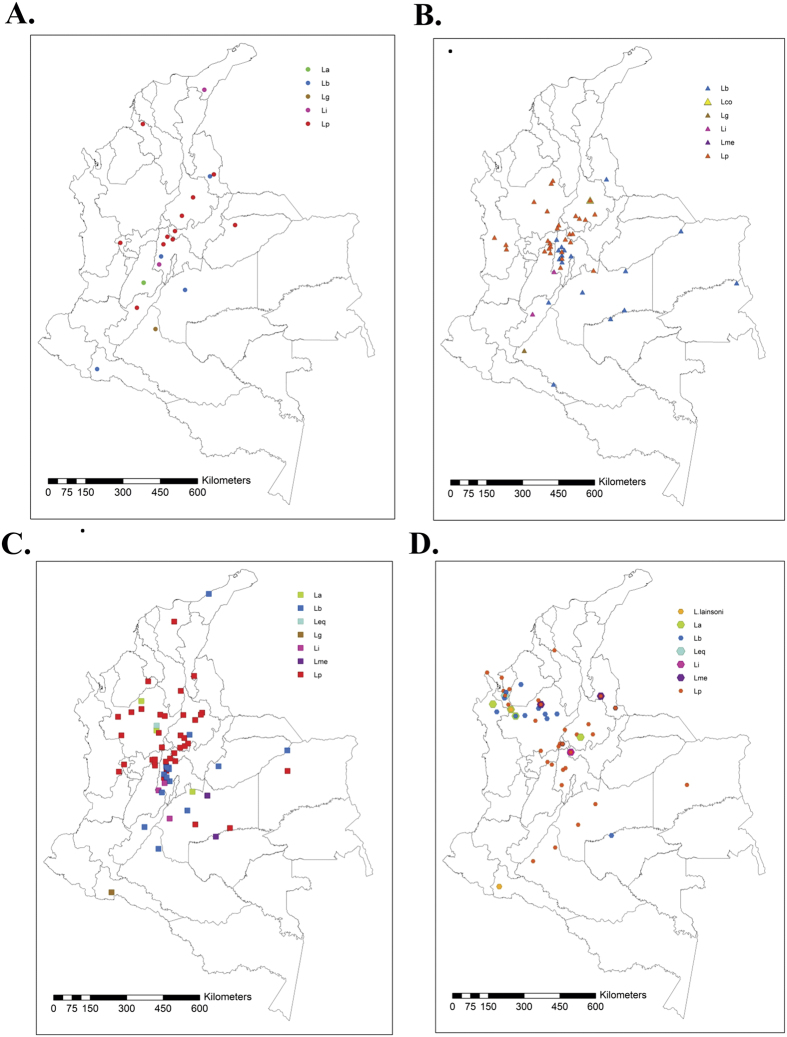
Temporal variation of *Leishmania* isolates from 1980 to 2001. Georeferenced isolates were built to construct maps in year ranges as follows. The maps were built on ArcGIS10.3 (http://www.esri.com/ArcGIS10.3) (**A**) 1990–1985; (**B**) 1986–1990; (**C**) 1991–1995; (**D**) 1996–2001.

In general, sampling was performed successively in all the departments during the time of the study, except for Bolivar, Magdalena and Sucre that were sampled once. From 1980 to 1986 six parasite species were detected in the country, mainly distributed in the Magdalena River Valley with most localities positive for *L. panamensis* in eight Departments. From 1986 to 1990 *L. braziliensis* was recorded in the Amazonia and Orinoquia regions while *L. panamensis* was detected in the north and west. In 1988 *L. colombiensis* was isolated from *P. shannoni*. From 1991 to 1995 *L. braziliensis* and *L. panamensis* were reported in new localities, with *L. panamensis* being found almost in every region of the country except the Amazonia, and *L. braziliensis* reaching the northern corner of Colombia in La Guajira. From 1996 to 2001, *L. lainsoni* was isolated for the first time in two spatially separated localities, Putumayo, in the south in 1997, and Antioquia in the north in 2001. *L. panamensis* was found in two new departments, Caquetá and Vichada in 1996 while *L. braziliensis* appeared for the first time in Cordoba in 1997 (Fig. 4).

Most collection localities (51%) belonged to Montane Forest coverage followed by moist forest (25%) and dry forest (16%); however, regarding land use coverages, 92% of the collection records were distributed in transformed ecosystems. It is important to note that collection data points did not belong to actual transmission sites thus further analyses could not be performed. Vector species present in all parasite isolation sites were *Psychodopygus panamensis, P. shannoni* and *Lutzomyia gomezi.* Isolation sites for *Leishmania amazonensis* furthermore coincided with *Nyssomyia yuilli yuilli, L. braziliensis* with *Pintomyia ovallesi,* and *L. mexicana* with *Nyssomyia antunesi.* Additionally, *L. infantum chagasi*, as expected for their proven association, was isolated in *Lu. longipalpis* distribution areas.

## Discussion

Despite the high number of cases annually occurring in Colombia, and the relevance of parasite species identification in the establishment of adequate management for Leishmaniases in Colombia, the surveillance systems have not developed an active and accurate strategy to control this pathology, as happens for many other neglected tropical diseases in Latin America. This premise is observed in the lack of updated studies regarding the epidemiology of the infection. Few studies have highlighted the over-representation of *L. panamensis, L. guyanensis* and *L. braziliensis* in humans, sandflies and mammalian reservoirs in Colombia^17^,^26^, and a retrospective study conducted in seven departments, detected the presence of *L. panamensis* (74,45%), *L. braziliensis* (15,33%), *L. guyanensis* (0,73%), *L. mexicana* complex (3,65%) and *Leishmania mexicana* (5,11%) in 137 isolates^14^. The technique employed in those studies was MLEE, but direct sequencing of genetic markers was never performed so far. Herein, we conducted the first retrospective study in Colombia (including 22 departments) using Cytb barcoding, finding the existence of nine species associated with CL (Figs 1 and 3).

Genetic barcoding using Cytb or other genetic marker has been useful for *Leishmania* species identification. The WHO suggests MLEE as the ‘gold standard’, but this method is time consuming limiting its potential as a gold standard^27^. Recently, the use of PCR-RFLP directed to Heat Shock Protein genes has shown a reasonable potential for species typing^28^. Also, the use of HRM (High Resolution Melting) platforms has proven to be useful for this purpose^29^. However, all these methods present some drawbacks that gene sequencing can solve. Some authors have shown how useful the Cytb barcoding could be in clinical samples and isolates due to its high sensitivity, specificity and be an uniparental informative^22^,^30^,^31^. In our case, the direct sequencing of Cytb allowed the discrimination of nine *Leishmania* species, just in the case of *L. braziliensis* we had to submit the samples to confirmation by HSP70 sequencing due to the equal identity percentage of *L. braziliensis* and *L. peruviana* in GenBank. Nevertheless, the congruence between Cytb barcoding and MLEE profiling was high (KI = 0.833 p < 0.05), the incorrect assignment was observed for *L. colombiensis, L. equatoriensis*, *L. mexicana* and *L. lainsoni*, this must have occurred due to the inability of MLEE to discriminate closely related species within the *Viannia* subgenus. Also, another explanation could be the likely existence of interspecies hybrids but we would need to conduct phylogenetic analyses with several molecular markers to prove this hypothesis. One vestige of this premise is that Corredor *et al.*, in 1980 reported a low percentage of isolates with ‘uncertain identity’ by MLEE means, which could have been the species herein reported. Our results show the high potential in the use of Cytb for species barcoding as has been demonstrated in Argentina where a high concordance was also observed^23^. However, it is well known that parasite isolation is not always successful and the distribution may have been biased because the analyses were only performed on isolates. New studies using clinical samples, insect vectors and samples from reservoirs are required to have an unbiased picture of the true distribution of *Leishmania* species in the country.

One of the advantages about using gene sequencing is the understanding of the intra-specific genetic diversity of *Leishmania* species. Our attempt was to describe the species circulating in the country but we could also determine its genetic diversity based on one single gene marker (Table 1; Fig. 2). All of the studies reported in Colombia have just shown the species distribution but never its diversity. In Brazil and Argentina a series of study using Multilocus Sequence Typing have been published but mainly focused on *L. braziliensis*^32^,^33^. MLST approaches have been conducted in Old World species as *L*. donovani, *L. infantum chagasi*, *L. tropica,* and *L. major* but not systematically in New World *Leishmania* species^8^,^34^,^35^,^36^. Our data supports interesting findings regarding a high genetic diversity displayed by *L. panamensis* (Hd = 0.123; S = 80). An important point to address is the high frequency of mutations in a conserved marker (Cytb) due to its uniparental inheritance status, therefore if examining single copy housekeeping genes the diversity may be greater than expected. In the haplotype network is observed that most of the isolates cluster in one dense haplotype (Fig. 2A). We just mentioned the case of *L. panamensis* due to the absence of MLST studies within this species but when examining *L. braziliensis* we observed a similar pattern (Hd = 0.390; S = 5). In the case of the haplotype network retrieved from *L. braziliensis* strains, two clear and dense haplotypes are tailored, one mainly disperse in the Andean region and the second across the Orinoquia and Atlantic region. This could provide insights about parasite genotype dispersal but future studies using high-resolution markers are required to fulfill these statements. This clearly suggests the need to apply MLST markers within *L. panamensis* and *L. braziliensis* in Colombia in order to understand the transmission dynamics and the plausible events of clonality and recombination across our dataset as discussed by different authors^37^,^38^.

Isolates from sandflies and mammals resulted according to previously published results and contributes new evidence of vector-parasite associations in Colombia. *Pintomyia spinicras*sa is a proven vector of *L. braziliensis* in Colombia and Venezuela, and was found infected with that parasite in one collection site in 1985^39^,^40^. *Psathyromyia shannoni* is a suspected vector of *L. braziliensis* in Bolivia, and was found infected with *Leishmania spp*. parasites in Colombia^40^. Our study provides new findings of *L. colombiensis* and *L. braziliensis* isolated from three individuals of this vector species in Santander. Interestingly, *Lu. longipalpis* was found infected with three parasite species (*L. infantum chagasi, L. braziliensis* and *L. panamensis*) in the same collection site in 1998 and 1992 suggesting vector permissivity. This vector species is suspected vector of *L. braziliensis*, *L. amazonensis* and *L. mexicana* in Brazil^41^,^42^.

*Leishmania equatoriensis* was originally reported in *Choloepus hoffmani* and *Sciurus granatensis* from Ecuador^43^ but not reported in humans so far. In this study we detected the parasite in two human isolates in 1995 from different localities in Antioquia where the highest number of CL cases is reported and six *Leishmania* species are known to occur (Fig. 3). This data need to be treated cautiously because a recent report argues about the true status of *L. equatoriensis* and *L. hertigi* as *Leishmania* species^3^. *Leishmania lainsoni* was also detected form two isolates; the geographical distribution of this species includes Brazil, Peru and Bolivia, and has been isolated from humans, sandflies and mammalian reservoirs^44^,^45^. Recent descriptions incriminate *Nyssomyia umbratilis* as the vector and a different range of mammals such as *Agouti paca, Cebus paella* and *Mesocricetus auratus* as reservoirs for this species^44^,^46^. Likewise, frequent mixed infections with *L. braziliensis* in humans with CL are reported in Peru and Bolivia. In Colombia, the sandfly *N. umbratilis* is present but probably remained undetected due to the low resolution of the methods employed in the past^40^.

Overall, we observed that *L. panamensis* and *L. braziliensis* are the most frequent species associated with CL in Colombia as previously reported^14^,^26^. Mucosal leishmaniasis (MCL) is also present in the country but we were not able to disregard if this clinical manifestation was present after isolation and patient follow-up. We also observed that *L. mexicana, L. amazonensis, L. guyanensis* and *L. infantum chagasi* are incriminated with CL (Fig. 1). Curiously, the autochthonous species *L. colombiensis* also reported in Venezuela and Panama was not detected in humans but detected in the sandfly *P. shannoni*. Laboratory studies indicate that at least three species of *Leishmania* parasites: *L. mexicana*, *L. panamensis* and *L. infantum chagasi* can develop in *P. shannoni*^47^, which is known to feed on mammals, including humans, and has been reported to transmit visceral leishmaniasis in dogs, hamsters, and other mammals. This would be the first report of natural infection in *P. shannoni* since previous reports show *Lutzomyia hartmanni* and *Lutzomyia gomezi* as vectors of this species^48^,^49^,^50^.

Regarding mammal species infected with *Leishmania* parasites, few studies on reservoir species have been conducted in Colombia^51^,^52^. This study revealed the presence of *L. panamensis* and *L. amazonensis* infecting dogs in two localities in the inter-Andean valleys. Dogs can get infected with the same parasite species, and show lesions similar to humans, but their role as reservoirs is discussed, and they are preferentially considered accidental hosts^53^,^54^. In our study, parasites were also isolated from synanthropic mammals such as *Didelphis marsupialis* infected with *L. infantum chagasi* and the rodent *Akodon* infected with *L. braziliensis. Didelphis marsupialis* is a known reservoir of *L. infantum chagasi* in Colombia^55^ and according to Brandao-Filho and collaborators, who found one *Akodon arviculoides* infected with *L. braziliensis* in Brazil, opposums and rodents constitute *L. braziliensis* reservoirs in Colombia and the Brazilian Amazonia^56^.

When the geographical distribution of *Leishmania* species was depicted, we did not detect a geographical clustering of the species as suggested by some authors in Colombia, but a broad distribution that seemed to increase during the time of the study^14^. We found that *L. panamensis* is broadly distributed in the country but *L. braziliensis* was more widespread (Fig. 3). Although distribution of *Leishmania* in an ecological context is variable and parasite dispersal includes migration of sandflies and reservoirs, parasite successive adaptations to new vector species and blood sources (Man/domestic animals), the broad pattern observed in this study could suggest that human migration can be highly affecting parasite species dispersal^57^,^58^. The constant human displacement in Colombia due to violence and political instability could be favoring the wider distribution of the different species. Recently, migration of *L. guyanensis* from the Amazonia forest, with new vector-parasite associations, was detected in two different habitats: the Andean valleys located above 1000 meters in the municipality of Chaparral (Tolima) and Sucre in the Colombian Caribbean coast^14^. The understanding of the ecology of leishmaniasis has to be proven from a continental level. It is interesting to observe the species distribution of *Leishmania* in neighbor countries as Ecuador, Peru and Venezuela. In Ecuador, *L. panamensis, L. guyanensis, L. braziliensis, L. mexicana, L. amazonensis* and *L. equatoriensis* have been described. In Peru, there exist reports of *L. peruviana, L. lainsoni, L. amazonensis, L. guyanensis* and *L. braziliensis*. Lastly, in Venezuela reports of *L. braziliensis, L. colombiensis, L. venezualensis, L. amazonensis, L. panamensis, L. garnhami, L. infantum chagasi* and *L. guyanensis*^2^. This broad geographical view supports our findings of new species circulating in Colombia, due to likely migrations of humans, insect vector or reservoirs from neighbor countries^59^.

In conclusion, we were able to discriminate nine *Leishmania* species isolated from cases of CL in Colombia from 1980 to 2001. Herein, we reported two new species circulating in the country and confirm the predominance of *L. panamensis* and *L. braziliensis* as the agents of CL in the country. We also showed the potential of the use of Cytb barcoding for species assignment and the need to pursue MLST studies to untangle the genetic diversity of an unknown species as *L. panamensis* and to contribute further information about *L. braziliensis*. These results are of paramount importance for the epidemiological surveillance of CL in the country and highlight the need to convince the Colombian government about the need to deploy and standardize tools for the species identification to provide adequate management to individuals suffering this pathology.

## Additional Information

**How to cite this article**: Ramírez, J. D. *et al.* Taxonomy, diversity, temporal and geographical distribution of Cutaneous Leishmaniasis in Colombia: A retrospective study. *Sci. Rep.* **6**, 28266; doi: 10.1038/srep28266 (2016).

## Supplementary Material

Supplementary Information

## Figures and Tables

**Table 1:** Genetic diversity parameters of 327 *Leishmania* Cytb gene sequences.

Species	N	S	Eta	Hd	π	θ	k
*L. panamensis*	201	80	85	0.123	0.00304	0.03981	1.036
*L. braziliensis*	88	5	5	0.39	0.00104	0.00243	0.433
*L. guyanensis*	3	1	1	0.667	0.00139	0.00139	0.667
*L. lainsoni*	2	16	16	1	0.03347	0.03347	16
*L. amazonensis*	9	4	4	0.643	0.00247	0.00323	1.179
*L. infantum chagasi*	12	10	10	0.318	0.00374	0.00742	1.667
*L. mexicana*	8	0	0	0	0	0	0
*L. colombiensis*	2	2	2	0.5782	0.00213	0.00213	10
*L. equatoriensis*	2	0	0	0	0	0	0

N = Number of sequences.

S = Number of polymorphic sites.

Eta = Total number of mutations.

Hd = Haplotype diversity.

K = Average number of nucleotide differences.
