# Supplementary material for: Taxonomy, diversity, temporal and geographical distribution of Cutaneous Leishmaniasis in Colombia: A retrospective study
Source: Sci Rep. 2016 Jun 22;6:28266. doi: 10.1038/srep28266 (PMC4916406; doi:10.1038/srep28266)
Supplement: Supplementary Information [file srep28266-s1.doc]

| **Isolate**  **Taxonomy, diversity, temporal and geographical distribution of Cutaneous Leishmaniasis in Colombia: A retrospective study**  Juan David Ramírez, Carolina Hernández^1^, Cielo M. León, Martha S. Ayala, Carolina Flórez, Camila González  Table S1. Database of the *Leishmania* isolates employed in this study | **Cyt B** | **Host** | **Municipality** | **Department** | **LAT** | **ALT** |
| --- | --- | --- | --- | --- | --- | --- |
| 26 | Lp | Human | San Pablo de Borbur | Boyaca | 05°39′47″N | 74°03′48″O |
| 46 | Lp | Human | Land zuri | Norte de Santander | 06°13′06″N | 73°48′40″O |
| 74 | Lp | Human | Utica | Cundinamarca | 05°11′14″N | 74°28′52″O |
| 80 | Lb | Human | Granada | Meta | 03°32′19″N | 73°42′02″O |
| 87 | Li | Human | Tocaima | Cundinamarca | 04°27′30″N | 74°38′04″O |
| 94 | Li | Human | San Juan del Cesar | Guajira | 10°46′16″N | 73°00′11″O |
| 95 | Lb | Human | Arboledas | Norte de Santander | 07°38′32″N | 72°47′58″O |
| 96 | Lp | Human | Pueblo Rico | Risaralda | 05°14′18″N | 76°02′11″O |
| 118 | Lp | Human | Yacop¡ | Cundinamarca | 05°27′34″N | 74°20′18″O |
| 125 | Lp | Human | Ovejas | Sucre | 09°31′57″N | 75°13′26″O |
| 127 | Lp | Human | Paz de Ariporo | Casanare | 05°52′50″N | 71°53′31″O |
| 133 | Lb | Insect | Arboledas | Norte de Santander | 07°38′32″N | 72°47′58″O |
| 148 | Lm | Human | Puerto Lleras | Meta | 03°01′20″N | 73°24′16″O |
| 155 | Lp | Human | Durania | Norte de Santander | 07°42′47″N | 72°39′27″O |
| 172 | Lp | Human | Paime | Cundinamarca | 05°21′49″N | 74°08′45″O |
| 178 | Li | Human | San Vicente de Chucur¡ | Santander | 06°52′52″N | 73°24′35″O |
| 188 | Lp | Human | San Vicente de Chucur¡ | Santander | 06°52′52″N | 73°24′35″O |
| 222 | La | Reservoir | Coyaima | Tolima | 03°47'51''N | 75°11'38''O |
| 415 | Lp | Human | San Roque | Antioquia | 06°29′06″N | 75°01′11″O |
| 464 | Li | Insect | Ricaurte | Cundinamarca | 04°16′51″N | 74°45′53″O |
| 500 | Lco | Insect | San Vicente de Chucur¡ | Santander | 06°52′52″N | 73°24′35″O |
| 534 | Lb | Human | Mapirip n | Meta | 02°53′21″N | 72°07′32″O |
| 542 | Lb | Human | Puerto Trujillo | Meta | 4°18′51″N | [72°04′57″O](http://tools.wmflabs.org/geohack/geohack.php?language=es&pagename=Puerto_Gaitán&params=4.3141694444444_N_-72.0825_E_type:city) |
| 550 | Lp | Human | Victoria | Caldas | 05°18′59″N | 74°54′40″O |
| 556 | Lb | Human | Viot | Cundinamarca | 04°26′14″N | 74°31′18″O |
| 561 | Lp | Human | Urab | Antioquia | 06°49′42″N | 75°30′47″O |
| 562 | Lp | Human | Samana | Caldas | 05°24′45″N | 74°59′32″O |
| 563 | Lb | Human | Guaduas | Cundinamarca | 05°04′01″N | 74°35′42″O |
| 568 | Lp | Human | Villahermosa | Tolima | 05°01′50″N | 75°06′58″O |
| 569 | Lp | Human | Mariquita | Tolima | 05°11′56″N | 74°53′35″O |
| 571 | Lb | Human | Caicar | Venezuela | 07°39′00″N | 66°10′00″O |
| 575 | Lb | Human | Guayabal de S¡quima | Cundinamarca | 4°52’52”N | 74°28’10”O |
| 577 | Lb | Human | Guayabal de S¡quima | Cundinamarca | 4°52’52”N | 74°28’10”O |
| 582 | Lb | Human | Mapirip n | Meta | 02°53′21″N | 72°07′32″O |
| 583 | Li | Reservoir | Ricaurte | Cundinamarca | 04°16′51″N | 74°45′53″O |
| 584 | Lb | Human | San Jos‚ del Guaviare | Guaviare | 02°34′22″N | 72°38′45″O |
| 592 | Lp | Human | Mariquita | Tolima | 05°11′56″N | 74°53′35″O |
| 593 | Lp | Human | Mariquita | Tolima | 05°11′56″N | 74°53′35″O |
| 594 | Lp | Human | El Bagre | Antioquia | 07°35′34″N | 74°48′31″O |
| 608 | Lp | Human | Puerto Boyac | Boyaca | 05°58′33″N | 74°35′18″O |
| 609 | Lp | Human | Puerto Boyac | Boyaca | 05°58′33″N | 74°35′18″O |
| 611 | Lb | Human | La Dorada | Caldas | 05°27′14″N | 74°39′53″O |
| 615 | Lp | Human | Condoto | Choco | 05°06’01”N | 76°32’44”O |
| 616 | Lp | Human | Anolaima | Cundinamarca | 4º 45´ 48´´N | 74°27´53´´O |
| 617 | Lb | Human | San Jos‚ del Guaviare | Guaviare | 02°34′22″N | 72°38′45″O |
| 618 | Lb | Human | San Jos‚ del Guaviare | Guaviare | 02°34′22″N | 72°38′45″O |
| 620 | Lb | Human | Anolaima | Cundinamarca | 4º 45´ 48´´N | 74°27´53´´O |
| 621 | Lb | Human | Nocaima | Cundinamarca | 05°04′11″N | 74°22′41″O |
| 622 | Lp | Human | San Rafael | Santander | 06°22′21″N | 73°14′00″O |
| 623 | Lp | Human | Victoria | Caldas | 05°18′59″N | 74°54′40″O |
| 625 | Lp | Human | Mariquita | Tolima | 05°11′56″N | 74°53′35″O |
| 626 | Lp | Human | Falan | Tolima | 05°08′00″N | 74°57′00″O |
| 627 | Lp | Human | Falan | Tolima | 05°08′00″N | 74°57′00″O |
| 628 | Lp | Human | Paime | Cundinamarca | 05°21′49″N | 74°08′45″O |
| 629 | Lp | Human | Land zuri | Santander | 06°13′06″N | 73°48′40″O |
| 630 | Lp | Human | San Pablo de Borbur | Boyaca | 05°39′47″N | 74°03′48″O |
| 631 | Lp | Human | Otanche | Boyaca | 5°39′28″N | 74°10′50″O |
| 632 | Lb | Human | Villeta | Cundinamarca | 05°00′32″N | 74°28′20″O |
| 633 | Lp | Human | San Vicente de Chucur¡ | Santander | 06°52′52″N | 73°24′35″O |
| 635 | Lp | Human | Yacop¡ | Cundinamarca | 05°27′34″N | 74°20′18″O |
| 637 | Lp | Human | Paime | Cundinamarca | 05°21′49″N | 74°08′45″O |
| 638 | Lb | Human | Maimache | Guain¡a | 03°52′15″N | 67°55′16″O |
| 639 | Lb | Human | Mundoviejo | Huila | 03°10′24″N | 74°57′57″O |
| 640 | Lb | Human | Granada | Meta | 03°32′19″N | 73°42′02″O |
| 641 | Lp | Human | Yacop¡ | Cundinamarca | 05°27′34″N | 74°20′18″O |
| 646 | Lp | Human | Zaragoza | Antioquia | 07°29′23″N | 74°52′09″O |
| 647 | Lp | Human | Falan | Tolima | 05°08′00″N | 74°57′00″O |
| 650 | Lp | Human | Tad¢ | Choco | 05°15′58″N | 76°33′54″O |
| 651 | Lp | Human | La Paz | Santander | 06°10′43″N | 73°35′22″O |
| 652 | Lp | Human | Armero | Tolima | 04°58′00″N | 74°54′00″O |
| 655 | Lp | Human | San Pablo de Borbur | Boyaca | 05°39′47″N | 74°03′48″O |
| 657 | Lp | Human | Victoria | Caldas | 05°18′59″N | 74°54′40″O |
| 659 | Lp | Human | Yacop¡ | Cundinamarca | 05°27′34″N | 74°20′18″O |
| 660 | Lp | Human | Viot | Cundinamarca | 04°26′14″N | 74°31′18″O |
| 661 | Lp | Human | Puerto Triunfo | Antioquia | 05°52′21″N | 74°38′25″O |
| 662 | Lb | Human | Puerto Legu¡zamo | Putumayo | 00°11′36″S | 74°46′55″O |
| 663 | Lb | Human | Villeta | Cundinamarca | 05°00′32″N | 74°28′20″O |
| 666 | Lb | Human | Utica | Cundinamarca | 05°11′14″N | 74°28′52″O |
| 669 | Lb | Human | San Juan de Rioseco | Cundinamarca | 04°51′04″N | 74°07′34″O |
| 670 | Lb | Human | La Mesa | Cundinamarca | 04°37′49″N | 74°27′45″O |
| 671 | Lp | Human | Alto Baud¢ | Choco | 05º31'33''N | 76º59'42''O |
| 674 | Li | Reservoir | Teruel | Huila | 02°44′31″N | 75°34′03″O |
| 675 | Lb | Human | Aguazul | Casanare | 5°10’22´´N | 72°32’49´´O |
| 676 | Lp | Human | Yacop¡ | Cundinamarca | 05°27′34″N | 74°20′18″O |
| 677 | Lb | Human | Utica | Cundinamarca | 05°11′14″N | 74°28′52″O |
| 678 | Lb | Human | Quipile | Cundinamarca | 04°44′53″N | 74°33′47″O |
| 682 | Lp | Human | Simit¡ | Bolivar | 07°57′28″N | 73°56′37″O |
| 683 | Lp | Human | Yacop¡ | Cundinamarca | 05°27′34″N | 74°20′18″O |
| 684 | Lp | Human | Saman | Caldas | 05°24′45″N | 74°59′32″O |
| 685 | Lb | Human | San Vicente del Cagu n | Caqueta | 02°07′18″N | 74°45′58″O |
| 687 | Lp | Human | Fundaci¢n | Magdalena | 10°31′14″N | 74°11′06″O |
| 688 | Lp | Human | Cimitarra | Santander | 06°18′51″N | 73°56′59″O |
| 690 | Lb | Human | San Antonio del Tequendama | Cundinamarca | 04°37′08″N | 74°21′14″O |
| 691 | Lp | Human | Santa Helena del Op¢n | Santander | 06°20′24″N | 73°37′01″O |
| 692 | Lp | Human | Mariquita | Tolima | 05°11′56″N | 74°53′35″O |
| 693 | Lp | Human | Otanche | Boyac | 5°39′28″N | 74°10′50″O |
| 698 | Lp | Human | Villeta | Cundinamarca | 05°00′32″N | 74°28′20″O |
| 699 | Lp | Human | Ayapel | Cordoba | 8°18′45″N | 75°08′42″O |
| 701 | Lp | Human | Yacop¡ | Cundinamarca | 05°27′34″N | 74°20′18″O |
| 702 | Lp | Human | Land zuri | Santander | 06°13′06″N | 73°48′40″O |
| 703 | Lb | Human | San Jos‚ del Guaviare | Guaviare | 02°34′22″N | 72°38′45″O |
| 704 | Lp | Human | Puerto valdivia | Antioquia | 07°17′16″N | 75°23′40″O |
| 705 | Lp | Human | Paime | Cundinamarca | 05°21′49″N | 74°08′45″O |
| 706 | Lp | Human | Paime | Cundinamarca | 05°21′49″N | 74°08′45″O |
| 707 | Lp | Human | Paime | Cundinamarca | 05°21′49″N | 74°08′45″O |
| 708 | Lp | Human | Cimitarra | Santander | 06°18′51″N | 73°56′59″O |
| 709 | Lp | Human | Yacop¡ | Cundinamarca | 05°27′34″N | 74°20′18″O |
| 715 | Lb | Human | La Mesa | Cundinamarca | 04°37′49″N | 74°27′45″O |
| 716 | Lp | Human | Segovia | Antioquia | 07°04′48″N | 74°41′56″O |
| 718 | Lp | Human | Pueblo Rico | Risaralda | 05°14′18″N | 76°02′11″O |
| 719 | Lp | Human | Pueblo Rico | Risaralda | 05°14′18″N | 76°02′11″O |
| 720 | Lp | Human | Pueblo Rico | Risaralda | 05°14′18″N | 76°02′11″O |
| 722 | Lp | Human | Victoria | Caldas | 05°18′59″N | 74°54′40″O |
| 723 | Lp | Human | La Belleza | Santander | 05°51′27″N | 73°57′56″O |
| 724 | Lb | Human | Puerto L¢pez | Meta | 04°05′04″N | 72°57′21″O |
| 725 | Li | Insect | Ricaurte | Cundinamarca | 04°16′51″N | 74°45′53″O |
| 727 | Lp | Human | Puerto L¢pez | Meta | 04°05′04″N | 72°57′21″O |
| 730 | Lp | Human | San Vicente de Chucur¡ | Santander | 06°52′52″N | 73°24′35″O |
| 731 | Lb | Human | Puerto Alonso. | Meta | [4°05′23″N](http://tools.wmflabs.org/geohack/geohack.php?language=es&pagename=Puerto_López&params=4.0897222222222_N_-72.961944444444_E_type:city) | 72°57′43″O |
| 734 | Lb | Human | Puerto Lleras | Meta | 03°01′20″N | 73°24′16″O |
| 736 | Lb | Human | Granada | Meta | 03°32′19″N | 73°42′02″O |
| 737 | Lg | Human | Puerto As¡s | Putumayo | 00°30′48″N | 76°30′03″O |
| 738 | Lp | Human | Remedios | Antioquia | 07°01′51″N | 74°32′00″O |
| 739 | Lb | Human | Nimaima | Cundinamarca | 05°07′34″N | 74°23′06″O |
| 740 | Lp | Human | Sucre | Santander | 05°55′06″N | 73°47′28″O |
| 744 | Lp | Human | Cimitarra | Santander | 06°18′51″N | 73°56′59″O |
| 745 | Lp | Human | Taraz | Antioquia | 07°35′01″N | 75°24′02″O |
| 748 | Lp | Human | Taraz | Antioquia | 07°35′01″N | 75°24′02″O |
| 749 | Lp | Human | Taraz | Antioquia | 07°35′01″N | 75°24′02″O |
| 750 | Lp | Human | Taraz | Antioquia | 07°35′01″N | 75°24′02″O |
| 751 | Lp | Human | San Jos‚ del Palmar | Choco | 04°58′27″N | 76°13′42″O |
| 755 | Lp | Human | Taraz | Antioquia | 07°35′01″N | 75°24′02″O |
| 756 | Lb | Human | Nocaima | Cundinamarca | 05°04′11″N | 74°22′41″O |
| 757 | Lp | Human | Taraz | Antioquia | 07°35′01″N | 75°24′02″O |
| 758 | Lb | Human | San Jos‚ del Guaviare | Guaviare | 02°34′22″N | 72°38′45″O |
| 759 | Lb | Human | Tobia | Cundinamarca | 05°07′36″N | 74°27′13″O |
| 760 | Lb | Human | Vian¡ | Cundinamarca | 04°52′26″N | 74°33′45″O |
| 761 | Lp | Human | Otanche | Boyaca | 5°39′28″N | 74°10′50″O |
| 768 | Lm | Human | Puerto L¢pez | Meta | 04°05′04″N | 72°57′21″O |
| 769 | Lb | Human | Trujillo | Vichada | 05°45′37″N | 70°00′29″O |
| 770 | Lb | Human | Anolaima | Cundinamarca | 4°45´48´´N | 74°27´53´´O |
| 773 | Lp | Human | Remedios | Antioquia | 07°01′51″N | 74°32′00″O |
| 776 | Lp | Human | Puerto Valdivia | Antioquia | 07°17′16″N | 75°23′40″O |
| 778 | Lp | Human | Taraz | Antioquia | 07°35′01″N | 75°24′02″O |
| 780 | Lp | Human | San Vicente de Chucur¡ | Santander | 06°52′52″N | 73°24′35″O |
| 785 | Lp | Human | Land zuri | Santander | 06°13′06″N | 73°48′40″O |
| 792 | Lp | Human | Rionegro | Santander | 07°09′13″N | 73°09′13″O |
| 795 | Lp | Human | El Carmen | Norte de Santander | 08°30′46″N | 73°27′03″O |
| 796 | Lp | Human | Bucaramanga | Santander | 7°04′43″N | 73°11′50″O |
| 800 | Lp | Human | Pradera | Caldas | 05°25′28″N | 74°53′48″O |
| 915 | Lp | Human | San Ignacio | Santander | 06°19′18″N | 73°20′48″O |
| 916 | Lp | Human | Sons¢n | Antioquia | 05°42′38″N | 75°18′38″O |
| 984 | Lp | Human | Puerto Berrío | Antioquia | 06°29′30″N | 74°24′12″O |
| 985 | Lp | Human | Cimitarra | Santander | 06°18′51″N | 73°56′59″O |
| 986 | Lp | Human | Cimitarra | Santander | 06°18′51″N | 73°56′59″O |
| 990 | Lp | Human | Cimitarra | Santander | 06°18′51″N | 73°56′59″O |
| 991 | Lp | Human | Cimitarra | Santander | 06°18′51″N | 73°56′59″O |
| 643A | Lp | Human | Aspirado - Otanche | Boyaca | 5°39′28″N | 74°10′50″O |
| 643B | Lp | Human | Otanche | Boyaca | 5°39′28″N | 74°10′50″O |
| 750A | Lp | Human | Tarazá | Antioquia | 07°35′01″N | 75°24′02″O |
| 784A | Lp | Human | Cachira | Norte de Santander | 7°44’27´´N | 73°02’55´´O |
| CL 001 | Lp | Human | Mariquita | Tolima | 05°11′56″N | 74°53′35″O |
| 82 | Lg | Human | San Vicente del Cagu n | Caqueta | 02°07′18″N | 74°45′58″O |
| 146 | Lb | Human | Durania | Norte de Santander | 07°42′47″N | 72°39′27″O |
| 245 | Lg | Human | Bel‚n de los Andaqu¡es | Caqueta | 01°25′06″N | 75°52′39″O |
| 280 | Lp | Human | Zaragoza | Antioquia | 07°29′23″N | 74°52′09″O |
| 466 | Li | Insect | Ricaurte | Cundinamarca | 04°16′51″N | 74°45′53″O |
| 495 | Lp | Human | Paime | Cundinamarca | 05°21′49″N | 74°08′45″O |
| 512 | Lb | Human | Puerto Gait n | Meta | 04°18′48″N | 72°04′54″O |
| 521 | Lp | Human | Victoria | Caldas | 05°18′59″N | 74°54′40″O |
| 522 | Lb | Human | Anolaima | Cundinamarca | 04°45′48″N | 74°27′53″O |
| 535 | Lb | Human | San Juan de Rioseco | Cundinamarca | 04°51′04″N | 74°07′34″O |
| 560 | Lp | Human | Cimitarra | Santander | 06°18′51″N | 73°56′59″O |
| 570 | Lb | Human | Quipile | Cundinamarca | 04°44′53″N | 74°33′47″O |
| 574 | Lp | Human | Villahermosa | Tolima | 05°01′50″N | 75°06′58″O |
| 672 | Lb | Human | Vian¡ | Cundinamarca | 04°52′26″N | 74°33′45″O |
| 747 | Lp | Human | Urrao | Antioquia | 06°18′55″N | 76°08′03″O |
| 765 | Lb | Human | Caparrap¡ | Cundinamarca | 5°20’39´´N | 74°29’30´´O |
| 788 | Lp | Human | Land zuri | Santander | 06°13′06″N | 73°48′40″O |
| 789 | Lp | Human | Rionegro | Santander | 07°09′13″N | 73°09′13″O |
| 797 | Lp | Human | San Vicente de Chucur¡ | Santander | 06°52′52″N | 73°24′35″O |
| 805 | Lp | Human | Cimitarra | Santander | 06°18′51″N | 73°56′59″O |
| 851 | Lb | No hay dato | Ituango | Antioquia | 07°10′16″N | 75°45′51″O |
| 881 | Lp | Human | Dabeiba | Antioquia | 07°00'05''N | 76°15'40''N |
| 902 | Lp | Human | Landazur¡. | Santander | 06°13′06″N | 73°48′40″O |
| 904 | Lp | Human | Landazur¡. | Santander. | 06°13′06″N | 73°48′40″O |
| 910 | Lp | Human | Cerro Yoqu¡. Necocl¡. | Antioquia | 08°25′33″N | 76°47′02″O |
| 919 | Lp | Human | Ver.Miramar Correg. San Jose de Apartad¢ | Antioquia | 07°25′45″N | 75°18′26″O |
| 931 | Lp | Human | Capurgan | Choco | 08°37′00″N | 77°20′00″O |
| 990 | Lp | Human | Cimitarra | Santander | 06°18′51″N | 73°56′59″O |
| 994 | Lg | Human | Orito | Putumayo | 00°40′36″N | 76°52′38″O |
| 1008 | La | Human | Mutatá | Antioquia | 07°14′39″N | 76°26′08″O |
| 1013 | Lp | Human | Carepa | Antioquia | 07°45′29″N | 76°39′19″O |
| 1015 | Lb | Human | Dabeiba | Antioquia | 07°00'05''N | 76°15'40''O |
| 1017 | Lb | Human | Chigorodó | Antioquia | 07°40′11″N | 76°40′53″O |
| 1020 | Lb | Human | Mutatá | Antioquia | 07°14′39″N | 76°26′08″O |
| 1021 | Lb | Human | Mutatá | Antioquia | 07°14′39″N | 76°26′08″O |
| 1023 | Lb | Human | Saiza | Cordoba | [8°10′22″N](http://tools.wmflabs.org/geohack/geohack.php?language=es&pagename=Tierralta&params=8.1727777777778_N_-76.059444444444_E_type:city) | 76°03′34″O |
| 1025 | Lb | Human | Apartado | Antioquia | 07°52′59″N | 76°37′33″O |
| 1031 | Lp | Human | Centro Calderón - Puerto Boyacá | Boyaca | 5°57'51.27"N | 74°29'17.4" |
| 1032 | Lp | Human | Centro Calderón - Puerto Boyacá | Boyaca | 5°57'51.27"N | 74°29'17.4"O |
| 128 | Lb | Human | San Mart¡n | Peru | 12°02′00″S | 76°43′00″O |
| 223 | Lp | Reservoir | Palermo | Huila | 02°53′30″N | 75°26′15″O |
| 224 | Lp | Reservoir | Palermo | Huila | 02°53′30″N | 75°26′15″O |
| 248 | Lb | Reservoir | Arboledas | Norte de Santander | 07°38′32″N | 72°47′58″O |
| 287 | Lme | Human | Ricaurte | Cundinamarca | 04°16′51″N | 74°45′53″O |
| 415 A | Lp | Human | San Roque | Antioquia | 06°29′06″N | 75°01′11″O |
| 444 | Lp | Human | Chaparral | Tolima | 04°19′51″N | 73°17′03″O |
| 452 | Lp | Human | San Vicente de Chucur¡ | Santander | 06°52′52″N | 73°24′35″O |
| 462 | Lp | Insect | Ricaurte | Cundinamarca | 04°16′51″N | 74°45′53″O |
| 463 | Lb | Insect | Ricaurte | Cundinamarca | 04°16′51″N | 74°45′53″O |
| 464 | Lb | Insect | Ricaurte | Cundinamarca | 04°16′51″N | 74°45′53″O |
| 834 | Lp | Human | Velez | Santander | 06°00′48″N | 73°40′25″O |
| 500 | Lco | Insect | San Vicente de Chucur¡ | Santander | 06°52′52″N | 73°24′35″O |
| 520 | Li | Human | Victoria | Caldas | 05°18′59″N | 74°54′40″O |
| 523 | Lp | Human | In¡rida | Guain¡a | 03°52′15″N | 67°55′16″O |
| 531 | Lb | Human | Quipile | Cundinamarca | 04°44′53″N | 74°33′47″O |
| 544 | Lb | Human | San Alberto | Cesar | 07°45′40″N | 73°23′32″O |
| 545 | Lp | Human | Victoria | Caldas | 05°18′59″N | 74°54′40″O |
| 547 | Lp | Human | Victoria | Caldas | 05°18′59″N | 74°54′40″O |
| 555 | Lp | Human | Victoria | Caldas | 05°18′59″N | 74°54′40″O |
| 559 | Lp | Human | Victoria | Caldas | 05°18′59″N | 74°54′40″O |
| 567 | Lp | Human | Mariquita | Tolima | 05°11′56″N | 74°53′35″O |
| 619 | Lp | Human | Anolaima | Cundinamarca | 4º 45´ 48´´N | 74º 27´ 53´´O |
| 740 | Lp | Human | Sucre | Santander | 05°55′06″N | 73°47′28″O |
| 742 | Li | Human | Anapoima | Cundinamarca | 4°33´13´´N | 74°32´22´´O |
| 766 | Li | Human | La Uribe,La Macarena y R¡o Guayabero. | Meta | 03°14′26″N | 74°21′15″O |
| 767 | Lp | Human | Barrancabermeja | Santander | 7°04′03″N | 73°50′50″O |
| 772 | Lp | Human | Caparrap¡ | Cundinamarca | 5°20’39´´N | 74°29’30´´O |
| 790 | Lb | Human | Land zuri | Santander | 06°13′06″N | 73°48′40″O |
| 794 | Lp | Human | Rionegro | Santander | 07°09′13″N | 73°09′13″O |
| 798 | Lp | Human | Land zuri | Santander | 06°13′06″N | 73°48′40″O |
| 673 | Lp | Human | Villeta | Cundinamarca | 05°00′32″N | 74°28′20″O |
| 804 | Lb | Human | Neiva | Huila | 02°55′38″N | 75°16′55″O |
| 850 | La | Human | Taraza | Antioquia | 07°35′01″N | 75°24′02″O |
| 830 | Lp | Human | Rionegro | Santander | 07°09′13″N | 73°09′13″O |
| 831 | Lb | Human | Bucaramanga | Santander | 7°04′43″N | 73°11′50″O |
| 818 | Lp | Human | La Belleza | Santander | 05°51′27″N | 73°57′56″O |
| 820 | La | Human | Bucaramanga | Santander | 7°04′43″N | 73°11′50″O |
| 822 | Lp | Human | Bucaramanga | Santander | 7°04′43″N | 73°11′50″O |
| 829 | Lb | Human | Rionegro | Santander | 07°09′13″N | 73°09′13″O |
| 821 | Lp | Human | San Vicente de Chucur¡ | Santander | 06°52′52″N | 73°24′35″O |
| 832 | Lb | Human | Santa Elena del Opon | Santander | 06°20′24″N | 73°37′01″O |
| 819 | Lb | Human | Melgar | Tolima | 04°12′17″N | 74°38′27″O |
| 853 | La | Human | San Jose del Nus | Antioquia | 06°29′40″N | 74°50′25″O |
| 878 | Leq | Human | Yal¡ | Antioquia | 06°40′28″N | 74°50′03″O |
| 847 | Lp | Human | Mapiripan | Meta | 02°53′21″N | 72°07′32″O |
| 855 | Lp | Human | Ituango | Antioquia | 07°10′16″N | 75°45′51″O |
| 879 | Lp | Human | Puerto Triunfo | Antioquia | 05°52′21″N | 74°38′25″O |
| 886 | Lp | Human | Caracol¡ | Antioquia | 06°24′26″N | 74°45′24″O |
| 892 | Lp | Human | Urab | Antioquia | 06°49′42″N | 75°30′47″O |
| 894 | Lp | Human | San Vicente del Cagu n | Caqueta | 02°07′18″N | 74°45′58″O |
| 884 | Lb | Human | Riohacha | Guajira | 11°32′40″N | 72°54′26″O |
| 899 | Lp | Human | Landazur¡. | Santander | 06°13′06″N | 73°48′40″O |
| 909 | Lp | Human | Vereda Nueva Antioquia. Turbo. | Antioquia | 07°59′52″N | 76°29′36″O |
| 911 | Lp | Human | San Jos‚ de Apartad¢. | Antioquia | 07°52′59″N | 76°37′33″O |
| 908 | Lp | Human | Vereda Nueva Antioquia. Turbo | Antioquia | 07°59′52″N | 76°29′36″O |
| 907 | Lp | Human | Vereda Miramar. Apartad¢. | Antioquia | 07°25′45″N | 75°18′26″O |
| 901 | Lp | Human | Landazur¡. | Santander | 06°13′06″N | 73°48′40″O |
| 905 | Lp | Human | Landazur¡. | Santander | 06°13′06″N | 73°48′40″O |
| 900 | Lp | Human | Landazur¡. | Santander | 06°13′06″N | 73°48′40″O |
| 912 | Lp | Human | Nueva Colonia. Turbo | Antioquia | 07°55′59″N | 76°42′59″O |
| 917 | Lp | Human | Sierra La Macarena | Meta | 2° 58′ 9″ N | 73° 54′ 2″ O |
| 913 | Lp | Human | Villeta | Cundinamarca | 05°00′32″N | 74°28′20″O |
| 922 | Lp | Human | Ver.Miramar. Correg. San Jos‚ de Apartad¢ | Antioquia | 07°25′45″N | 75°18′26″O |
| 925 | Lb | Human | El Dari‚n | Antioquia | 07°09′28″N | 76°58′15″O |
| 928 | Lp | Human | Nocaima | Cundinamarca | 05°04′11″N | 74°22′41″O |
| 923 | Li | Human | Otanche | Boyaca | 5°39′28″N | 74°10′50″O |
| 950 | Lb | Human | Anorí | Antioquia | 7°04´25´´N | 75°08´49´´O |
| 954 | Lb | Human | Amalfi | Antioquia | 6º54‘17”N | 75º04’36”O |
| 958 | Lp | Human | Marquetalia | Caldas | 05°17′48″N | 75°03′18″O |
| 955 | Lp | Human | Iquitos | Perú | 03°44′00″N | 73°15′00″O |
| 949 | Lp | Human | Puerto Berrío | Antioquia | 06°29′30″N | 74°24′12″O |
| 939 | Lp | Human | Puerto Boyacá | Boyac | 05°58′33″N | 74°35′18″O |
| 942 | Lp | Human | Mariquita | Tolima | 05°11′56″N | 74°53′35″O |
| 935 | Lp | Human | Labateca | Norte de Santander | 07°17′56″N | 72°29′41″O |
| 932 | Lp | Human | Mariquita | Tolima | 05°11′56″N | 74°53′35″O |
| 937 | Lb | Human | Labateca | Norte de Santander | 07°17′56″N | 72°29′41″O |
| 934 | Lb | Human | Labateca | Norte de Santander | 07°17′56″N | 72°29′41″O |
| 948 | Lb | Human | Peque | Antioquia | 07°01′16″N | 75°54′33″O |
| 936 | Lb | Human | Labateca | Norte de Santander | 07°17′56″N | 72°29′41″O |
| 957 | Lp | Human | Cumaribo | Vichada | 04°26′44″N | 69°47′56″O |
| 956 | Lb | Human | Guaviare | Guaviare | 02°34′22″N | 72°38′45″O |
| 982 | Lp | Human | Porroso (Uraba Antioqueño) | Antioquia | 07°25′21″N | 76°31′57″O |
| 991 | Lp | Human | Cimitarra | Santander | 06°18′51″N | 73°56′59″O |
| 987 | Lp | Human | Cimitarra | Santander | 06°18′51″N | 73°56′59″O |
| 984 | Lp | Human | Puerto Berrío | Antioquia | 06°29′30″N | 74°24′12″O |
| 993 | Lb | Human | Puerto Valdivia | Antioquia | 07°17′16″N | 75°23′40″O |
| 1006 | Lp | Human | Carepa | Antioquia | 07°45′29″N | 76°39′19″O |
| 1022 | La | Human | Dabeiba | Antioquia | 07°00'05''N | 76°15'40''N |
| 1019 | Lp | Human | Mutatá | Antioquia | 07°14′39″N | 76°26′08″O |
| 1004 | Lp | Human | Córdoba | Córdoba | 09°26′16″N | 74°47′43″O |
| 1007 | Lp | Human | Mutatá | Antioquia | 07°14′39″N | 76°26′08″O |
| 870 | Lp | Human | Landázuri | Santander | 06°13′06″N | 73°48′40″O |
| 1011 | Leq | Human | Carepa | Antioquia | 07°45′29″N | 76°39′19″O |
| 1005 | Lb | Human | Chigorodó | Antioquia | 07°40′11″N | 76°40′53″O |
| 1009 | Lp | Human | Mutatá | Antioquia | 07°14′39″N | 76°26′08″O |
| 1016 | La | Human | Riosucio | Choco | 07°26′25″N | 77°07′00″O |
| 1012 | Lp | Human | Dabeiba | Antioquia | 07°00'05''N | 76°15'40''N |
| 1014 | Lb | Human | Dabeiba | Antioquia | 07°00'05''N | 76°15'40''O |
| 1008 | Lp | Human | Mutatá | Antioquia | 07°14′39″N | 76°26′08″O |
| 1003 | Lp | Human | Caquetá | Caqueta | 01°37′00″N | 75°36′00″O |
| 1018 | Lp | Human | San José de Apartado (Antioquia) | Antioquia | 07°52′59″N | 76°37′33″O |
| 1026 | Lp | Human | Puerto Triunfo | Antioquia | 05°52′21″N | 74°38′25″O |
| 1040 | Lp | Human | Carmen de Chucurí | Santander | 06°41′53″N | 73°30′40″O |
| 1034 | Lb | Human | Centro Calderón - Puerto Boyacá (Boyacá) | Boyaca | 5°57'51.27"N | 74°29'17.4"O |
| 1030 | Lp | Human | Centro Calderón - Puerto Boyacá (Boyacá) | Boyaca | 5°57'51.27"N | 74°29'17.4"O |
| 992 | Lb | Human | Segovia | Antioquia | 07°04′48″N | 74°41′56″O |
| 920 | Lme | Human | Ver. Miramar Correg. San Jos‚ de Apartad¢ | Antioquia | 07°25′45″N | 75°18′26″O |
| 927 | Lp | Human | Viot | Cundinamarca | 04°26′14″N | 74°31′18″O |
| 897 | Lb | Human | San Jos‚ de Apartad¢, Urab . | Antioquia | 07°52′59″N | 76°37′33″O |
| 995 | Lg | Human | Mutatá | Antioquia | 07°14′39″N | 76°26′08″O |
| 854 | Lp | Human | Puerto Valdivia | Antioquia | 07°17′16″N | 75°23′40″O |
| 846 | Lme | Human | San Jose del Guaviare | Guaviare | 02°34′22″N | 72°38′45″O |
| 1024 | Lp | Human | Carepa (Antioquia) | Antioquia | 07°45′29″N | 76°39′19″O |
| 728 | Lp | Human | Quipile | Cundinamarca | 04°44′53″N | 74°33′47″O |
| 735 | Lb | Human | Granada | Meta | 03°32′19″N | 73°42′02″O |
| 765 | Lme | Human | Caparrap¡ | Cundinamarca | 5°20’39´´N | 74°29’30´´O |
| 764 | Lb | Human | Tobia | Cundinamarca | 05°07′36″N | 74°27′13″O |
| 781 | La | Human | Pi¤alito | Meta | 04°13′54″N | 73°30′29″O |
| 793 | Lp | Human | San Vicente de Chucur¡ | Santander | 06°52′52″N | 73°24′35″O |
| 533 | Lb | Human | Puerto Boyac | Boyac | 05°58′33″N | 74°35′18″O |
| 532 | Lp | Human | Granada | Meta | 03°32′19″N | 73°42′02″O |
| 874 | La | Human | Landázuri | Santander | 06°13′06″N | 73°48′40″O |
| 784A | Lme | Human | Cachira | Norte de Santander | 7°44’27´´N | 73°02’55´´O |
| 784B | Lp | Human | Cachira | Norte de Santander | 7°44’27´´N | 73°02’55´´O |
